# Advances in Molecular Profiling and Their Potential Influence on the Extent of Surgery in Well-Differentiated Thyroid Carcinoma (WDTC)

**DOI:** 10.3390/life13061382

**Published:** 2023-06-13

**Authors:** Constantinos Parpounas, Vasilis Constantinides

**Affiliations:** 1 Department of Endocrine Surgery, Evangelistria Medical Centre, 1 Michael Giorgalla Street, 1095 Nicosia, Cyprus; parpounas@gmail.com; 2 Medical School, University of Nicosia, 93 Ayiou Nikolaou Street, Engomi, 2408 Nicosia, Cyprus

**Keywords:** thyroid, cancer, molecular, genetics, surgery, TERT, BRAF

## Abstract

Thyroid cancer surgery has evolved dramatically with advances in our understanding of the biological behaviour of WDTC. Molecular profiling is shedding light on the subset that may behave aggressively. In an era when thyroid cancer management is becoming increasingly conservative, decision making regarding the extent of surgery must be objectively guided by molecular markers. The aim of the present article is to summarise the current published literature and provide possible practice recommendations. An online search for relevant published articles was performed using several databases. Title, abstract, and full-text screening, along with data extraction, was performed by two independent reviewers after the inclusion and exclusion criteria were defined. A total of 1241 articles were identified, and 82 relevant articles were extracted and scrutinised. BRAF V600E and TERT promoter mutations were found to be associated with an increased risk of disease recurrence and distant metastases. Several other mutations have been identified that enhance disease aggressiveness (such as RET/PTC, PTEN, and TP53). One of the most important determinants of the outcome in WDTC is the extent of surgical resection. The evolution of molecular testing has reached a stage of personalised incorporation into surgical practice. Guidelines for molecular testing and surgery in WDTC will need to be clearly defined, arguably representing the next chapter in the management of the disease.

## 1. Introduction

Thyroid cancer is the commonest endocrine malignancy and accounts for 3% of all cancers diagnosed worldwide every year, with an increasing incidence over the last decades [1]. Up to 95% are well-differentiated carcinomas (WDTC) derived from thyroid follicular cells, with up to 85% being of papillary (PTC) morphology, and up to 15% being of follicular (FTC) morphology. Most WDTCs are diagnosed at an early stage, with the majority having an excellent prognosis, with 10-year survival rates of over 90%; 20% of patients will, however, experience disease persistence of recurrence or even progression with distant metastases [2]. More conservative and personalized treatment options have been developed in recent years to face the rising diagnosis of low-risk WDTC [3], with hemithyroidectomy being proposed for tumours up to 4 cm in size, depending on pre-operative risk assessment [4]. Many important risk-stratification features, however, are frequently unknown pre-operatively (such as central compartment lymph node status, aggressive histological variants, and microscopic extrathyroidal extension). The presence of these features would upgrade the disease from low to intermediate or even high risk, with studies suggesting that pre-operative clinical findings only manage to identify 18% of patients with indications for total thyroidectomy, and up to 60% of patients end up requiring a completion thyroidectomy [5]. The inability to provide optimal surgery at the onset can be detrimental to a patient’s well-being, adding morbidity and cost and wasting limited and precious healthcare resources.

Molecular thyroid nodule testing has become widely available for cytologically indeterminate thyroid nodules so as to facilitate appropriate management decisions. The addition of similar molecular signature testing in cytologically proven WDTC is currently gaining acceptance as a valuable tool to aid correct pre-operative risk stratification and thus guide practitioners towards an optimal surgical strategy from the onset. There is increasing evidence that the use of these techniques may enhance the clinician’s desire to offer “personalised cancer medicine” [6]. The aim of the present paper is to present the current progress in the field, highlight controversies, and provide recommendations for current practice and future research.

## 2. Materials and Methods

### 2.1. Search Strategy

An online database search for relevant published articles was performed by two independent investigators (CP and VC) using the Cochrane Database of Systematic Reviews, PubMed, Embase, and Google. The last day of the search was the 15 November 2022. The following terms were searched: “molecular”, “genetic mutations”, “thyroid cancer”, “papillary thyroid carcinoma”, “follicular thyroid carcinoma”, and “well differentiated thyroid cancer”. Combination searches of the above-mentioned key terms were performed, and the two independent reviewers (CP and VC) extracted and evaluated the relevant articles to formulate the review.

### 2.2. Eligibility Criteria and Study Selection

Original articles, meta-analyses, and systematic reviews on human adults in the English language were included. References of included articles were also scanned to further enhance the accuracy of the search. Case reports, experimental articles (on nonhuman subjects), duplicates, articles that included only paediatric thyroid cancer, and articles not in English language were excluded.

### 2.3. Data Extraction

The data extracted from each study included the authors’ names and institutions, patient demographics, publication year, country, distribution of WDTC, prognostic data, treatment strategies, follow-up, specific mutations and pathways analysed, and their relevance to the prognosis and surgical strategy, both in terms of locoregional and distant metastases.

### 2.4. Analysis

A narrative review and the interpretation of relevant data were performed. No statistical analysis was performed.

## 3. Results

From the review of the literature, 1241 articles were identified, and 82 relevant articles were extracted and scrutinized. Data were retrieved from a combination of these articles and enriched as necessary with further in-depth study of their references. All articles used are presented in serial fashion in the following sections.

### 3.1. Molecular Pathogenesis of WDTC

**Figure 1:**
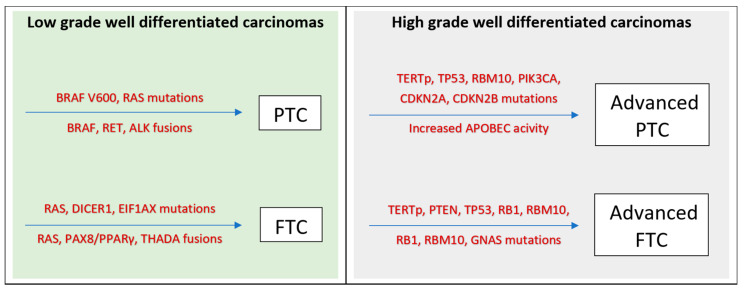
Genetic evolution of low-grade, well-differentiated carcinomas to high-grade, well-differentiated carcinomas. Abbreviations: PTC: papillary thyroid cancer; FTC: follicular thyroid cancer.

Molecular analysis of *The Cancer Genome Atlas (TCGA)* identified molecular alterations in 97% of the studied tumours [7,8]. These seem to be concentrated in a limited number of genes and frequently occur in a mutually exclusive manner. The most affected pathways were involved with mitogen-activating protein kinase (MAPK and BRAF alterations) and phosphatidylinositol-3-kinase (PI13K/AKT and RAS alterations). Other mutations, such as RET/PTC rearrangement, activate both pathways, while other less common mutations affect completely distinct pathways (PAX8/PPARγ). These are considered primary-driver mutations that lead to carcinogenesis, with other mutations occurring downstream that seem to confer biological aggressiveness to WDTC (e.g., TERTp, TP53, PTEN, and others; see Figure 1) [9]. PTCs, follicular adenomas, and minimally invasive FTCs from the TCGA cohort were classified into BRAF V600E-like, RAS-like, and non-BRAF/non-RAS types with different biological behaviours [10].

### 3.2. Specific Mutations and Associations with Prognosis

#### 3.2.1. BRAF Mutations

BRAF is a serine–threonine kinase, a key enzyme in the MAPK intracellular pathway that is key to cell growth, proliferation, apoptosis, and differentiation. BRAF mutations potently activate this pathway and may be considered driver lesions in thyroid carcinogenesis. BRAF V600E is the most frequent point mutation in PTC [11,12], and its incidence varies substantially among different geographical regions (up to 90% in Asia, with an average of 45% for PTC) [13,14,15,16,17]. BRAF mutations are rare in follicular thyroid carcinoma (1.4%), and they do not seem to exist in benign nodules. The specificity of BRAF V600E in PTC specimens is 100% [18,19], but they have a median sensitivity of only 30% [20]. In a next-generation sequencing analysis of a large cohort of advanced WDTC, BRAF mutations were found in 74% of PTC and 7.7% of FTC [21]. The prognostic value of BRAF V600E seems to be inconsistent in the literature published in English. Several studies relate BRAF V600E with recurrence, lymph node metastasis (LNM), extrathyroidal extension, and loss of radioiodine avidity [22,23,24,25,26,27], even in the setting of micropapillary or low-risk intrathyroidal carcinoma (T1–2, N0, and M0) [28], while other studies do not independently associate tumour aggressiveness with the presence of the mutation alone, especially after the clinicopathological characteristics are considered [29,30]. Furthermore, long-term, disease-specific survival (over 5 years) was not found to be affected by BRAF mutations in two published studies [31,32].

More recent studies suggest synergistic effects with additional mutations, such as TERT promoter (TERTp) mutation, resulting in increased tumour invasion and a poor prognosis [33,34,35,36,37].

BRAF K601E is a rarer mutation, with a low specificity to cancer (mostly follicular-variant PTC), thus carrying a relatively good prognosis [22,38,39] compared to BRAF V600E mutation. BRAF K601E can also be related to follicular adenoma [22,38].

#### 3.2.2. RAS

RAS mutations are relatively common mutations in PTC and are by far the commonest in FTC (66% in advanced disease) [21]. These mutations result in the constitutive, unregulated activation of GTP. The commonest mutations in thyroid cancer are found in NRAS and are present in both PTC and follicular carcinomas (FTC) in 6–20% of patients [40,41] and in 40–50% of patients [41], respectively. They are targets of the PI3K/AKT signalling pathway. Despite an association with tumour dedifferentiation and more aggressive behaviour [41], they are considered as driver (precursor) lesions where further mutations are needed for carcinogenesis, especially in FTC and FVPTC [42]. There is also evidence to suggest the positive correlation of RAS mutations with distant metastatic disease and reduced survival in WDTC [43]. RAS mutations, however, are the primary alterations of benign NIFTP and follicular adenomas in up 67% and 25% of cases, respectively [41,44,45,46]. Contrary to BRAF V600E, which is 100% specific to thyroid cancer, the specificity of RAS mutations ranges from 74 to 88% [47].

#### 3.2.3. RET/PTC Rearrangement

The RET/PTC proto-oncogene encodes a tyrosine kinase receptor that is not usually expressed in thyroid follicular cells (but which is highly expressed in parafollicular C cells), but it can be activated by chromosomal rearrangement—the RET/PTC translocation. The incidence in sporadic PTC is variable, ranging from 7 to 20% [8,48]. They activate both MAPK and PI3K-AKT pathways and are 100% specific to PTC [47]. RET/PTC translocations have been associated with a younger age at diagnosis and with a high propensity for nodal metastases [49]. RET/PTC1 and RET/PTC3 are the commonest rearrangements [8,50]. RET/PTC1 is found in 60–70% of cases [41] and associated with more indolent tumours when comparing with RET/PTC3 that has been associated with radiation-induced tumours [51]. Nevertheless, patients with RET/PTC translocations appear to be radioactive iodine avid [52].

#### 3.2.4. PAX8/PPAR Translocation

PAX/PPARγ fusion rearrangement has an inactivating effect on the tumour-suppressor gene PPARγ and is found in 30–60% of FTC and in 38% of the follicular variant of papillary thyroid cancer (FVPTC) [42,44]. They are generally seen in small tumours in relatively young patients, with frequent vascular invasion [41]. There is no overlap with RAS mutations within the same tumour, possibly suggesting distinct oncogenic pathways [44]. It has also been suggested that additional mutations are required, such as DICER, PIK3CA, and PTEN, for a follicular adenoma to be transformed to FTC [34,53].

#### 3.2.5. TERT

Relatively recently, human telomerase reverse transcriptase promoter (TERTp) gene mutations were found to be associated with advanced and aggressive thyroid cancers [54]. TERTp mutations, and more specifically C228T and C250T, account for 5–25% of PTC [12,55,56,57,58,59,60] and 35% of FTC [55,56]. It is speculated that it causes higher telomerase activity, resulting in aggressive PTCs [59,60,61,62]. The next-generation sequencing of advanced WDTCs revealed TERTp mutations to be second only to BRAF mutations in frequency, with a presence in 61% of advanced PTCs and in 71% of advanced FTCs [21]. Several articles associate TERTp mutations with poor prognosis, tumour invasion, and decreased survival [58,59,61,62,63,64,65]. A synergistic effect between TERTp and BRAF V600E or RAS mutations has been well-documented by several studies and has been shown to promote tumour aggressiveness and negatively impact disease-free survival [34,35,36,57,64,66,67,68,69,70]. BRAF and TERTp mutations are currently included in the risk stratification of locoregional recurrence in the 2015 *American Thyroid Association (ATA) Guidelines for DTC* [3]. The presence of TERTp mutations, with or without BRAF mutations, has been shown to confer a risk of cancer recurrence of >40% [3].

#### 3.2.6. PLEKHS1

The PLEKHS1 promoter mutation is relatively rare and is only detected in 1% of PTCs [70]. On the other hand, PLEKHS1 mRNA overexpression always occurs in PTCs [70]. In cases where lymph node (LN) infiltration is present, the level of PLEKHS1 mRNA is significantly higher when compared to PTCs without LN metastasis. In patients with distant metastases, the overexpression of PLEKHS1 mRNA was even more prominent [70]. PLEKHS1 expression has not been found to be associated with reduced overall survival. However, the co-existence of TERTp mutations is associated with poorer overall survival [61]. The utility of this molecular marker is still limited, and it is undergoing further research.

#### 3.2.7. Other Mutations

Several other mutations are described in the literature. Mutations in tumour-suppressor genes have been found in 20% of advanced PTCs. Furthermore, several co-existing tumour-suppressor gene mutations (PTEN, TP53, and others) have been found in advanced follicular thyroid carcinomas [21]. PTEN mutations in isolation have been found in 2% of advanced PTCs and in 14% of advanced FTCs. TP53 mutations have been found in up to 12% of advanced WDTCs [71].

### 3.3. Molecular Testing Kits and Usage in Current Practice

The diagnosis of indeterminate thyroid nodules (ITNs) based on the Bethesda System for Reporting Thyroid Cytopathology (BSRTC) ranges from 15 to 30% [72,73]. The risk of malignancy (ROM) for ITNs ranges from 10 to 40% when NIFTP is considered benign [74]. When diagnostic surgery is offered for ITNs, 10–40% of those patients are diagnosed with thyroid cancer [75]. The ultrasonographic features of have ITN with EU-TIRADS 2 have a ROM of less than 10%, while those with EU-TIRADS 4/5 have a ROM as high as 79% [76,77,78,79,80]. Molecular tests aim to refine the BSRTC-based inconclusive results by reclassifying the ITNs, aiming to avoid unnecessary diagnostic lobectomies and two-stage thyroidectomies. Moreover, attempts have been made to predict the preoperative risk of cancer recurrence based on molecular alterations.

The Afirma gene sequencing classifier (GSC), the successor of the Afirma gene expression classifier (GEC), is a “rule out” test with a sensitivity of 91% and a negative predictive value (NPV) of 96% for ITN [81]. The validation study illustrated that it has a specificity of 68% and a positive predictive value (PPV) of 47% [81]. Moreover, GSC demonstrates a 5-fold increase in specificity for Hurthle cell tumours [81], with subsequent studies confirming the initial results [82,83,84]. In addition, the negative results of GCS from ITN resulted in a decrease of diagnostic lobectomies from 74% to 7.6% [85,86]. The Afirma Xpression Atlas (XA) is a complementary 593-gene panel for GSC, enabling the detection of genomic variants and the further classification of suspicious GSC results, aiming to increase the overall PPV. Despite the improvement in detecting additional genomic alterations, it does not include the detection of TERTp mutations [87].

ThyGeNEXT/ThyraMIR is a dual-platform test, based on next-generation sequencing, which utilizes both mutational and micro RNA markers [88,89]. It offers 95% sensitivity, 90% NPV, 90% specificity, and 75% PPV for ITN, classifying it as a “rule in” and “rule out” test for ITN according to a retrospective multicentre cohort study [88]. Most common mutations and fusions associated with thyroid cancer are included. BRAF V600E, TERTp, RET/PTC, and others, as well as BRAF- and RET-related fusions, are categorized as strong driver mutations. The reasoning is based on either their BRAF-V600E-like behaviour, high PPV and association, or high risk for aggressive disease [55,56,62,65,90,91,92,93]. RAS-like mutations (such as NRAS, KRAS, KRAS, and PAX8/PPARγ) are categorised as weak driver mutations. ThyraMIR is utilized for RAS-like mutations, minimally invasive FTCs, low-grade PTCs, and Hurthle cell nodules to better differentiate nodules with a high risk of malignancy, thus improving diagnostic accuracy [89].

ThyroSeq v3 (TSv3) utilizes the next-generation sequencing of DNA and RNA from 112 genes and currently boasts the largest prospective, multicentre, double-blinded validation study of any commercially available test [94]. The study concluded that TSv3 can be considered as a “rule in” and “rule out” test, having a 94% sensitivity, 97% NPV, 82% specificity, and 66% PPV. It could also help to reduce diagnostic lobectomies by 61%. Additional studies confirmed the abovementioned results [95,96,97]. TSv3 divides test results into two categories, negative or positive, based on the probability of cancer or NIFTP [94]. Positive samples are further analysed to establish the risk of cancer recurrence and to subdivide them into low-, intermediate-, and high-risk groups. RAS-like alterations (RAS, PAX/PPARγ, and BRAF K601E) are categorized as comprising a low-risk group. BRAF-V600E-like alterations are categorized as having an intermediate risk of recurrence. TERTp mutations, particularly when found in combination with either RAS-like or BRAF-V600E-like alterations; TP53; AKT1; and PIK3CA mutations are categorized in the high-risk group [98].

Comparative studies between molecular diagnostic tests are limited in number. A parallel, prospective, randomised trial from a single centre examined the performance of TSv3 and GCS. The results were very similar between those two tests and lacked any statistically significant difference in sensitivity, specificity, NPV, and PPV. They both offered a similar percentage of thyroid-surgery avoidance: 49% (TSv3) and 51% (GCS) [99].

## 4. Discussion

Controversy exists when it comes to the extent of surgery for differentiated thyroid cancer, with conflicting data in the published literature [100,101]. There is a continuous shift towards more conservative surgery, even for WDTCs deemed pre-operatively to be “low-risk” and up to 4 cm in size [4]. The onus is currently on the clinician to consider all available pre-operative elements and offer the optimal form of treatment to carefully selected patients. Maintaining the balance between operative morbidity (in cases of disease overtreatment) and re-operative scenarios (in cases of initial undertreatment or persistent/recurrent disease) is difficult and frequently based on incomplete information and a balance of probabilities. Molecular profiling of WDTCs is reaching a point where it may be utilised to facilitate evidence-based decision making at a genetic level, enabling the clinician to provide personalised treatment to patients.

Previous reports have attempted to provide guidance and incorporate molecular testing into clinical practice. Yip et al. proposed a model where pre-operative molecular testing on FNAC specimens for BRAF, RAS, PAX8/PPARγ, and RET-PTC significantly contributed to correct risk-stratification and appropriate initial extent of surgery [48]. Miccoli et al. proposed the incorporation of molecular testing where conventional clinical and radiological features are unable to confidently risk-stratify WDTCs [102]. In a 2022 review, Sipos et al. suggested that identification of “driver mutations” alone (such as BRAF V600E and RAS) was not recommended as a guide for initial therapeutic strategies [103]. Acknowledgment was, however, made of the TERTp mutation and its ability, together with a set of other biomarkers, to independently influence the extent of surgery. Furthermore, the current literature seems to favour a model of progression from follicular adenomas/NIFTP/low-risk carcinomas to high-risk, aggressive WDTCs with the accumulation of mutations in a combination of genes [104]. Regarding the latter, two very recent reviews indicate that TERTp mutations seem to play a central role in risk-stratification. (This mutation was not considered in previous literature examining the same issues.)

**Figure 2:**
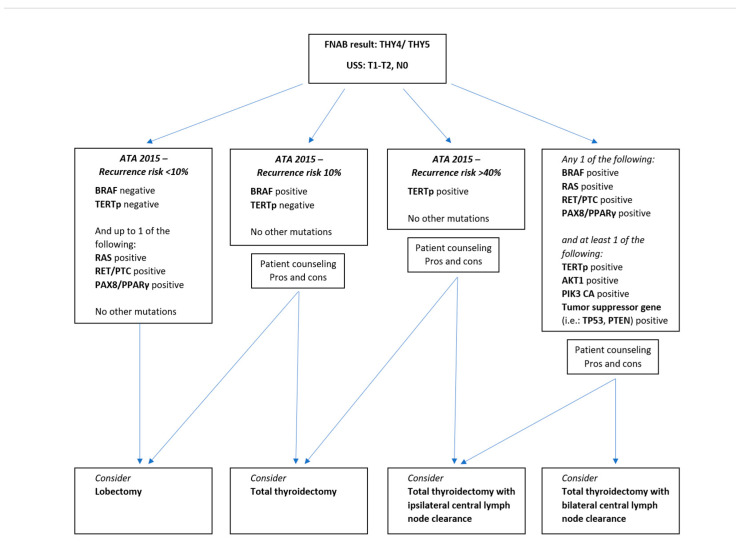
Clinical decision-making algorithm for patients with ultrasonographic and fine-needle aspiration biopsy results that are diagnostic or highly suspicious for well-differentiated thyroid cancers, assuming neither clinical nor radiological evidence of locoregionally advanced disease. Abbreviations: FNAB: fine-needle aspiration biopsy; THY4: suspicious for malignancy; THY5: malignant; USS: ultrasound scan; T1: tumour diameter of less than 2 cm without evidence of extrathyroidal extension; T2: tumour diameter between 2 and 4 cm without evidence of extrathyroidal extension; N0: without evidence of locoregional lymph node involvement; ATA: *American Thyroid Association Guidelines*; Pros: advantages; Cons: disadvantages.

Having analysed the current literature as related to the present manuscript, it is the belief of the authors that TERTp mutation seems to be instrumental in the shift from indolent to aggressive behaviour in WDTC, with other mutations having additional synergistic effects. TERTp, tumour-suppressor gene, PTEN, and several other mutations seem to confer a “second hit” effect after a driver mutation has occurred (such as BRAF or RAS), further driving the aggressive biological behaviour of WDTCs. The authors recognise that this position is clearly an oversimplification of what is obviously a very complex and evolving field. It is, however, made in light of the best available current evidence and may be able to steer clinical decision making and stimulate further research. The authors propose a clinical decision-making algorithm (Figure 2) that can be applied to patients with USS + FNAC, which are diagnostic or highly suspicious for WDTC, yet lacking clinical/radiological evidence of locoregionally advanced disease (T1–2 and N0). In proposing this algorithm, the authors acknowledge that proportionately more extensive operations may be performed on patients previously managed in a more conservative manner. A very important consideration is the potential increase in the incidence of postoperative complications, such as permanent hypoparathyroidism and recurrent laryngeal nerve injury [105,106]. A delicate balance needs to be maintained—avoiding as many re-operations as possible, while minimizing operative risk. The authors believe that a more conservative surgery should be proposed unless there is compelling evidence to suggest otherwise. Patient counselling should be at the forefront of this decision-making process, with patient preference given due consideration, especially in scenarios where a number of surgical options may be potentially applicable. In addition, to further minimize morbidity risk, operations over-and-above a total thyroidectomy should be performed in specialised endocrine surgery centres, as has been conclusively presented in several studies [107].

Limitations exist for the present study. The cost-effectiveness of performing molecular testing was not taken into consideration as no paper was found to directly analyse this aspect. There is evidence that molecular testing is cost-effective for ITNs [103], but no direct evidence applicable to WDTC. The authors recognise the significant cost of these tests, which must be incorporated into the setting of healthcare systems. The authors also recognise that, particularly for follicular lesions, the pre-operative diagnosis of carcinoma is very frequently impossible. The addition of molecular testing to this setting needs to be implemented selectively when suspicious clinical/radiological features exist, and in a similar manner to the well-studied methodology for ITNs. Furthermore, the authors recognise that it may be impossible to be exhaustive in extracting and analysing complex, evolving data such as those included in the present manuscript; minor omissions may have occurred that, however, are highly unlikely to alter the general concept and theme of the manuscript. The aim of the present paper is not to include every potential mutation that has been recognised to date, but rather to include well-studied pathways that can be applied clinically at the present time, without overcomplicating the decision making process.

## 5. Conclusions and Future Perspectives

The molecular profiling of WDTC is a rapidly evolving field, and it is one that holds great promise in individualising the management of this disease when used in conjunction with current, established criteria. TERTp seems to be at the centre of a series of aggression-conferring tumour behaviours. Further research will eventually enable molecular profiling to be fully incorporated into clinical practice in defining the extent of surgery for WDTC.

The future development of predictive models that will combine genetic data together with clinical and cytological findings will allow for accurate preoperative risk assessment, accurately guiding individualised treatment options. Furthermore, using liquid biopsy, circulating tumour cells, free nucleic acids, and tumour-derived extracellular vesicles released into the bloodstream can be detected in a noninvasive way and may further assist in the diagnosis, management, and prognosis of WDTC in the near future [108].

## Data Availability

No new data were created.
